# Evaluation of IsoCal geometric calibration system for Varian linacs equipped with on‐board imager and electronic portal imaging device imaging systems

**DOI:** 10.1120/jacmp.v15i3.4688

**Published:** 2014-05-08

**Authors:** Song Gao, Weiliang Du, Peter Balter, Peter Munro, Andrew Jeung

**Affiliations:** ^1^ Department of Radiation Physics The University of Texas MD Anderson Cancer Center Houston TX; ^2^ Varian Medical Systems Palo Alto CA USA

**Keywords:** IsoCal calibration, on‐board imager, quality assurance, imaging system

## Abstract

The purpose of this study is to evaluate the accuracy and reproducibility of the IsoCal geometric calibration system for kilovoltage (kV) and megavoltage (MV) imagers on Varian C‐series linear accelerators (linacs). IsoCal calibration starts by imaging a phantom and collimator plate using MV images with different collimator angles, as well as MV and kV images at different gantry angles. The software then identifies objects on the collimator plate and in the phantom to determine the location of the treatment isocenter and its relation to the MV and kV imager centers. It calculates offsets between the positions of the imaging panels and the treatment isocenter as a function of gantry angle and writes a correction file that can be applied to MV and kV systems to correct for those offsets in the position of the panels. We performed IsoCal calibration three times on each of five Varian C‐series linacs, each time with an independent setup. We then compared the IsoCal calibrations with a simplified Winston‐Lutz (WL)‐based system and with a Varian cubic phantom (VC)‐based system. The maximum IsoCal corrections ranged from 0.7 mm to 1.5 mm for MV and 0.9 mm to 1.8 mm for kV imagers across the five linacs. The variations in the three calibrations for each linac were less than 0.2 mm. Without IsoCal correction, the WL results showed discrepancies between the treatment isocenter and the imager center of 0.9 mm to 1.6 mm (for the MV imager) and 0.5 mm to 1.1 mm (for the kV imager); with IsoCal corrections applied, the differences were reduced to 0.2 mm to 0.6 mm (MV) and 0.3 mm to 0.6 mm (kV) across the five linacs. The VC system was not as precise as the WL system, but showed similar results, with discrepancies of less than 1.0 mm when the IsoCal corrections were applied. We conclude that IsoCal is an accurate and consistent method for calibration and periodic quality assurance of MV and kV imaging systems.

PACS numbers: 87.55.Qr, 87.56.Fc

## I. INTRODUCTION

Varian linear accelerators (linacs) with a kilovoltage (kV) on‐board imager (OBI) plus cone‐beam computed tomography (CBCT) and a megavoltage (MV) electronic portal imaging device (EPID) (Varian Medical Systems, Inc., Palo Alto, CA) are widely used for image‐guided radiotherapy (IGRT). The coincidence of the MV and kV imaging isocenters and the radiation treatment isocenter is essential for high‐precision, image‐guided radiotherapy. For a linac used for stereotactic radiosurgery (SRS) and/or stereotactic body radiation therapy (SBRT), the coincidence of the MV and kV imaging coordinate systems and the treatment coordinate system (for four cardinal angles) within ±1 mm is highly desired, while for other radiation therapies this coincidence should be within ±2 mm.^(1)^ Recently, Task Group 179 of the American Association of Physicists in Medicine (AAPM) recommended that the coincidence of the MV and kV imagers and room lasers should be within ±1 mm.^(2)^ Linacs equipped with kV OBI and MV EPID imaging systems have four isocenters to characterize: the mechanical isocenter, the radiation treatment isocenter, the kV imaging system isocenter, and the MV imaging system isocenter. The locations and sizes of these isocenters differ for various reasons such as gantry sag, uncertainty in the calibration of the imaging arms, and mechanical sag in the imaging arms. A highly accurate and efficient quality assurance (QA) system is required to calibrate or verify the coincidence of the MV and kV imager centers with the treatment isocenter for linacs with integrated EPID and OBI‐CBCT imaging systems. In addition, QA needs to be done on a regular basis^(1)^ to verify the alignment between these isocenters.

The Varian cubic phantom (VC) method^(3)^ is a common QA procedure used to compare the MV and kV isocenters with the mechanical isocenter in clinical practice. Other phantom‐based methods have also been proposed.^4^, ^5^, ^6^ In these methods, a phantom is aligned to the mechanical isocenter using room lasers or some other surrogate for the radiation treatment isocenter; therefore, the calibration accuracy relates to the accuracy of the chosen surrogate rather than directly to the treatment isocenter. These methods were reviewed by Bissonnette et al.^(7)^ and they found that the accuracy of geometric calibration of the OBI system was stable within 2 mm over 28 months.

Another widely used QA method, the Winston‐Lutz (WL) method,^(8)^ uses a small object (usually a metallic BB) that is fixed in room space. Images of the object at the isocenter are acquired using the treatment beam with a small predefined field at discrete gantry angles and are analyzed to find the treatment isocenter. This method was originally used as part of the patient setup for stereotactic radiosurgery, but has been adopted for other uses, such as verification of the kV and MV imaging systems. For the past several years, the WL method has been used as part of manufacturers' acceptance tests^(9)^ and for routine QA.^(10)^ Another approach is to use a cylindrical phantom with two rings of BBs for geometric calibration.^(11)^ This technique uses symmetry to create virtual points that are then used to eliminate dependencies between variables. The challenge is to identify the BBs uniquely, especially in an MV beam.

Varian introduced an automated geometric calibration system for OBI and EPID imaging systems called IsoCal as part of the TrueBeam platform. The IsoCal system^(12)^ quickly and precisely determines the locations of the treatment isocenter and the kV and MV imaging isocenters. A similar IsoCal system has been released for the existing Varian C‐series linacs. The theory of operation of the IsoCal system is not the focus of this work, but is presented in Appendix A.

In this study, we evaluated the IsoCal system with multiple Varian C‐series linacs equipped with OBI‐CBCT and EPID imaging systems. We compared the calibration results obtained using IsoCal with those obtained by the in‐house WL method^(10)^ and the VC method.^(3)^ The goal of this study was to assess the accuracy, consistency, and reproducibility of the IsoCal calibration system for calibration and verification of OBI and EPID imaging systems geometry. On the basis of our results, we implemented the IsoCal calibration method as a standard QA procedure for Varian C‐series linacs with OBI and EPID imaging systems.

## II. MATERIALS AND METHODS

### A. overview of the IsoCal system

**Figure 1:**
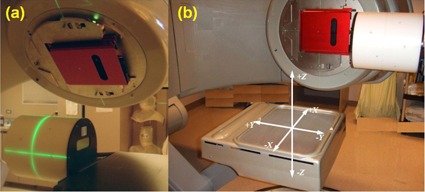
The IsoCal phantom, IsoCal collimator plate, and calibration setup (a); coordinate system used by IsoCal (b).

The purpose of the IsoCal system is to determine the treatment isocenter of the linac and to calculate image offsets for MV and kV images as a function of gantry angle so that the DICOM coordinates of these images are exactly aligned with the treatment isocenter. The IsoCal system (Fig. 1(a)) consists of a phantom, a collimator plate, and application software. The phantom is a hollow cylinder 23 cm in diameter and length with 16 tungsten‐carbide BBs (each 4 mm in diameter) located in a precisely known geometry on the surface (the IsoCal BB phantom). The collimator plate is an aluminum plate with a steel pin in its center. The plate attaches to an accessory slot of the MV collimator and has a spring‐loaded locking system to ensure that the plate will not move with respect to the collimator upon collimator or gantry rotation. The software consists of an application that runs on the OBI workstation and takes in DICOM format images of the phantom and collimator plate. The software uses these images to determine the location of the treatment isocenter and the distance between the treatment isocenter projection and the centers of the kV and MV images as a function of gantry angle.

The process of IsoCal calibration starts by acquiring images of the IsoCal BB phantom and collimator plate using the MV beam at four collimator angles (195°, 270°, 0°, 90°) while the gantry is fixed at 0°. Then, the collimator plate and phantom are imaged at eight gantry angles (225°, 270°, 315°, 0°, 45°, 90°, 135°, 180°) using the MV imaging system with the collimator fixed at 0°, and kV images of the phantom are acquired at the same gantry angles without moving the phantom. These images are loaded into the IsoCal software, and the software uses the MV images to determine the location of the treatment isocenter with respect to the phantom. Once this position is known, the software calculates offsets between the position of the imager panels and the treatment isocenter as a function of gantry angle. These offsets are used to create an XML file that contains corrections to the location of the imagers as a function of gantry angle. This file can be used by the OBI system to adjust the DICOM coordinates of acquired kV and MV images to better match the location of the treatment isocenter.^(12)^ These coordinates are used by both the internal matching system on the Varian 4D Integrated Treatment Console (4D ITC) and external systems, such as MOSAIQ (Elekta AB, Stockholm, Sweden), when the images are used for verification of patient setup. For CBCT, each individual projection is corrected before being used in the CT reconstruction and the kV imager rotation center is also corrected and used as a consistent location for image reconstruction. It should be noted that this procedure is different from that used by the IsoCal system on the TrueBeam platform. The TrueBeam uses the IsoCal data to apply physical corrections to the panel position during image acquisition, whereas the control system on the C‐series platform does not support these real‐time corrections to the imager positions.

### B. General procedures of IsoCal calibration

The first step of the IsoCal calibration process is the setup. The collimator plate is inserted into the slot of the collimator interface in the machine head, the phantom is mounted at the front end of the table, and the scribed marks in the phantom are aligned with the room lasers (Fig. 1(a)).

The second step is image acquisition. Three test plans that accompany the IsoCal software (in DICOM format) are used for acquiring MV and kV images of the phantom and collimator plate. Each plan has either MV or kV setup fields which contain collimator angles, gantry angles, and position information of the MV or kV imager panels. Each test plan is delivered exactly like an actual patient treatment, with the setup field mode up on the treatment workstation (4D ITC), which sends the data to the OBI workstation. The IsoCal correction application is turned off in OBI administration before the predefined plan is loaded in “DICOM RT” mode on the 4D ITC. MV images are acquired at four different collimator angles. Then, MV images are acquired at eight gantry angles at 45° intervals. Finally, kV images are acquired at eight source angles. All three sets of images are saved in the designated folders.

The third step is calibration. The calibration relies on imaging the phantom with a precisely known BB geometry while rotating the gantry completely around the phantom. Since the phantom has well‐known geometry, the 2D coordinates of the BBs in the acquired images can be predicted by knowing the nominal locations of the X‐ray source, phantom, and imager. Any deviations in the actual 2D coordinates of the BBs determined from the acquired images can be attributed to motion of the X‐ray source and/or imager. The general calibration process consists of determining the treatment isocenter, the phantom position, and the source‐to‐imager distance (SID), and then finding the offsets between the MV and kV image centers and the projected treatment isocenter at different gantry angles.

**Figure 2:**
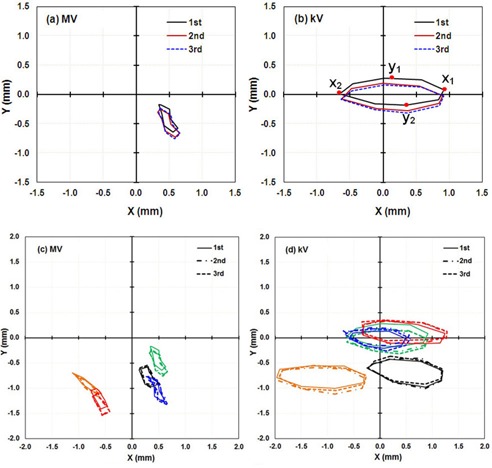
IsoCal results for three independent calibrations in a row for five linacs: the lateral (X) and longitudinal (Y) offsets in a full gantry rotation between the MV ((a) to (c)) and kV imager centers ((b) and (d)) (the origin) and the projected treatment isocenter. $X_{1}$ $X_{2}$, $Y_{1}$ and $Y_{2}$ are the points in each direction with the extreme values in that direction as illustrated in (b) (first curve); these extreme values were used as a metric of IsoCal reproducibility, as presented in Table 1. (a) and (b) are the results for linac M1, in (c) and (d): M1, Green, M2: Orange; M3: Red; M4: Black; M5: Blue. **Table 1:** Maximum differences ($\delta X_{1}$, $\delta X_{2}$, $\delta Y_{1}$, $\delta Y_{2}$) in extreme values (mm) for MV and kV panel corrections in each direction for the three IsoCal determinations for the five linacs. Figure 2 shows the full data for M1 and the definitions of $X_{1}$, $X_{2}$, $Y_{1}$, $\delta Y_{2}$. All corrections are given in the plane of the isocenter

As the IsoCal calibration is completed, the IsoCal software generates a review report of the calibration result on the screen, as well as a detailed result file in XML format that contains all the information about the image acquisitions, offsets between the treatment isocenter and the MV and kV imager centers for different gantry angles, and offsets between the phantom center and the treatment isocenter. These offsets are represented as 2D shift vectors (X, Y) with respect to source angle that indicate the lateral (X, along gantry rotation direction) and longitudinal (Y, perpendicular to gantry rotation) shifts needed for the MV and kV imager centers to align with the treatment isocenter^12^, ^13^ (Fig. 1(b)). The IsoCal software also generates extreme offsets $X_{1}$, $X_{2}$ and $Y_{1}$, $Y_{2}$ in both positive and negative directions relative to the MV and kV imager isocenters (see illustrations in Fig. 2). It should be noted that all IsoCal corrections are generated at the location of the imager panel (150 cm SID), and we have scaled all of these to the correction to isocenter (100 cm source‐to‐axis distance). This scaling was done to make comparisons with the WL and VC methods easier because those methods are stated at the isocenter. It also gives a better understanding of the clinical impact of these corrections.

	*MV*				*kV*			
	$\delta X_{1}$	$\delta X_{2}$	$\delta Y_{1}$	$\delta Y_{2}$	$\delta X_{1}$	$\delta X_{2}$	$\delta Y_{1}$	$\delta Y_{2}$
M1	0.0	0.1	0.2	0.1	0.0	0.0	0.2	0.2
M2	0.1	0.1	0.1	0.1	0.1	0.1	0.2	0.1
M3	0.1	0.1	0.2	0.1	0.0	0.1	0.1	0.0
M4	0.0	0.0	0.1	0.1	‐0.1	0.0	0.1	0.1
M5	0.1	0.1	0.1	0.1	0.2	0.2	0.2	0.1

The fourth step is applying IsoCal correction. When IsoCal correction is enabled in the OBI administration, the OBI and EPID imaging systems will correct the positions of acquired images using 2D shift vectors that describe the offsets between the MV and kV imager centers and the treatment isocenters.^(12)^

### C. Evaluation of reproducibility and robustness

#### C.1 Short‐term reproducibility

To test the short‐term reproducibility of the IsoCal system, a physicist performed the IsoCal calibration three times on the same day on the same linac. For each calibration, the phantom and collimator plate were set up independently from the previous setup, the OBI application and IsoCal software were restarted, and the imaging arms were retracted and reextended to ensure that there was no unintentional linkage between the calibrations. This reproducibility test was done on five different Varian C‐series linacs to look for machine dependencies. The IsoCal calibrations are stored as offset vectors (X, Y) in an XML file for the kV and MV imager panel locations. We compared the shape of the IsoCal correction curves versus gantry angle, as well as the extrema of the corrections in each direction for the three independent calibrations of each linac, and we also compared those offsets across five linacs ($M_{i}$, i=1,…,5).

#### C.2 Dependency of IsoCal results on phantom setup

To evaluate how phantom setup errors affected the calibration results, after the first calibration with optimal IsoCal phantom setup (set up to the room lasers per the manufacturer's instructions), we repeated the calibrations four times with different known offsets in the phantom position from the optimal position. For the first three trials, the phantom was shifted 5 mm in one direction at a time. For the last trial, the phantom was shifted 5 mm in each of the three directions (lateral, vertical, and longitudinal).

#### C.3 Dependency of IsoCal results on phantom set construction

To evaluate the effects of variations in phantom set (IsoCal phantom and the collimator plate) construction, we performed the IsoCal calibrations on the same linac (M1) five times in one day using five different IsoCal phantom sets. The IsoCal phantom sets were purchased in two batches, one was purchased about one year before the other four. It is assumed that all phantoms meet the manufacturer's internal quality control procedures.

### D. Independent evaluations of IsoCal calibration

Two different methods were used to measure the offsets between the MV and kV imager centers and the treatment or mechanical isocenter, and these methods were compared with the IsoCal calibrations. One method used the in‐house, WL‐based system,^(10)^ and the other used the VC‐based system.^(3)^

#### D.1 Tests using the in‐house WL method

Our in‐house, WL‐based system consists of a multileaf collimator (MLC) to define the radiation field, a metal BB held by a rigid plastic rod, and MATLAB‐based software (The MathWorks, Natick, MA). We set up the BB phantom close to the machine isocenter using room lasers; it is not necessary to place the BB phantom exactly at the machine isocenter.^(10)^ The BB was imaged with MV and kV X‐ray beams at each of the four gantry cardinal angles (0°, 90°, 180°, and 270°). The center of the MLC‐defined radiation field and the location of the BB were automatically determined in each of the MV images. Then the radiation treatment isocenter was determined at the intersection of the four radiation field centers. In each of the MV and kV images, the digital graticule was localized relative to the BB. The digital graticule is a structure superimposed on the image at the time of review that shows the location of the treatment isocenter as projected on the imager based on the DICOM coordinates of the imager position. Finally, the distance between the digital graticule and the treatment isocenter was derived at each of the four gantry cardinal angles. In this WL method, the BB position is not iteratively adjusted since it is used only as a reference point in the 3D space. In addition, the accuracy in localizing the treatment isocenter relies on the proper alignment of the MLC.

In the absence of IsoCal correction, the digital graticule in these images was located at the center of the image panel by default. When the IsoCal correction was applied, the correction to the digital graticule was read from the header of the DICOM image. The amount of correction in the imager plane was specified in the DICOM tag X‐Ray Image Receptor Translation.

For each linac, we performed one WL test and processed the resulting MV and kV images. The software computed the distances between the treatment isocenter and the digital graticules with and without the IsoCal correction. Thus, the WL method provided an independent evaluation of the effectiveness of IsoCal calibration.

#### D.2 Tests using the VC method

A VC‐based phantom was used to determine the offsets between the mechanical isocenters and the MV and kV imaging isocenters at the four cardinal gantry angles. The VC phantom provided by Varian is shaped as a cube^3^ and it was modified by adding tungsten wires to the surface. The wires on the VC phantom were aligned to the machine's mechanical isocenter using room lasers and/or the projection of the machine's light field crosshairs (the lasers were previously checked). After acquiring one MV (and kV) image at each cardinal source angle, the image window/level was adjusted so that we could visualize the wires on the surface of the cube, and the ruler tool in the OBI software was used to measure the distance from the center of the cube (image of the wires) to the center of the DICOM coordinates of the isocenter as projected on the imager panel (digital graticule).^(3)^ This procedure was done twice without moving the phantom, once with IsoCal corrections applied and once without IsoCal corrections.

## III. RESULTS

### A. IsoCal calibration precision

**Figure 3:**
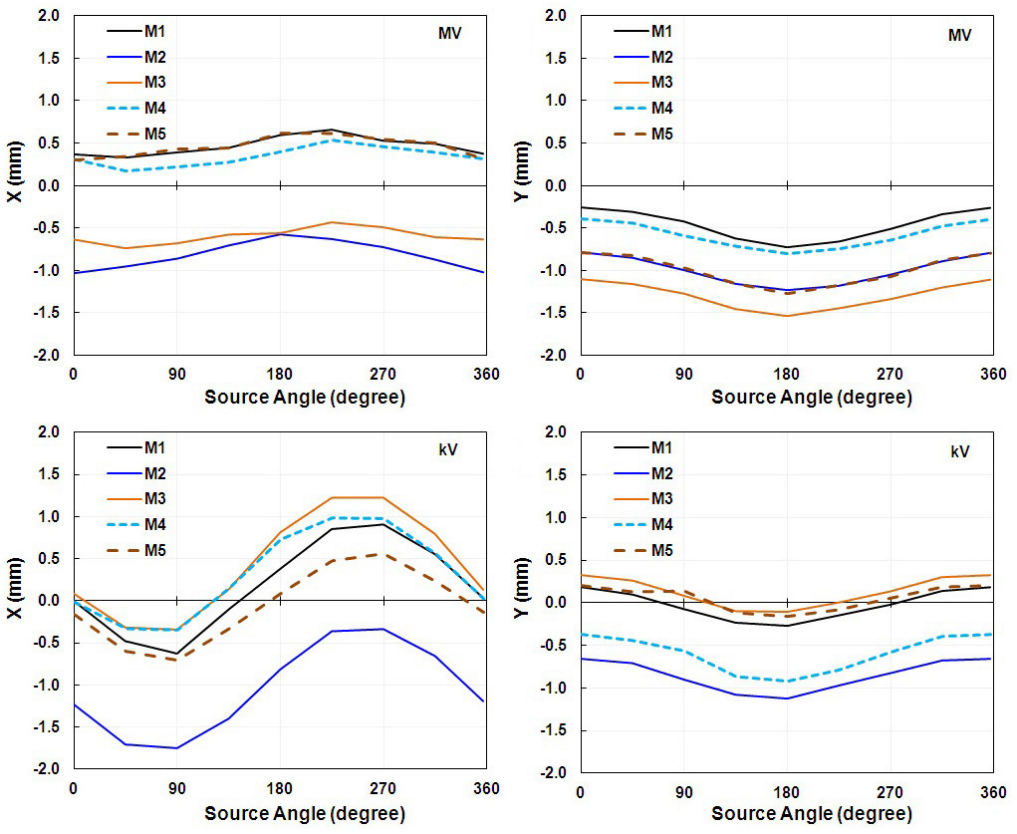
IsoCal results for all five linacs showing the offsets between the imager center and the projected treatment isocenter in the lateral (X) and longitudinal (Y) directions in a full gantry rotation for one calibration for the MV imager (upper graphs) and the kV imager (lower graphs).

We performed three independent IsoCal calibrations for each of five linacs. The three independent calibration results for linac M1 are shown as an (X, Y) correction for full gantry rotation in Fig. 2. We found that for all five linacs, the shapes of the corrections, as well as the extrema of the offsets between the MV and kV imager centers and the projected treatment isocenter, were very consistent across the three calibrations. The shape of the corrections indicates that the MV/kV images isocenter shift from the treatment isocenter as a function of gantry angle. Since the shape of the correction was consistent for repeated calibrations, we only present the maximum differences in the extrema of each set of three IsoCal determinations for the five linacs (Table 1). The maximum differences in the extrema of the three IsoCal determinations for all five linacs were within 0.2 mm for both the MV imager and the kV imager. Since the variations between the three IsoCal results were very small, we plotted the corrections of only one of the three IsoCal calibrations in the × and y panel directions versus source angle (Fig. 3). We noted that the largest corrections were 1.5 mm for the MV and 1.8 mm for the kV imagers across the five linacs.

### B. Setup uncertainty tolerance

**Figure 4:**
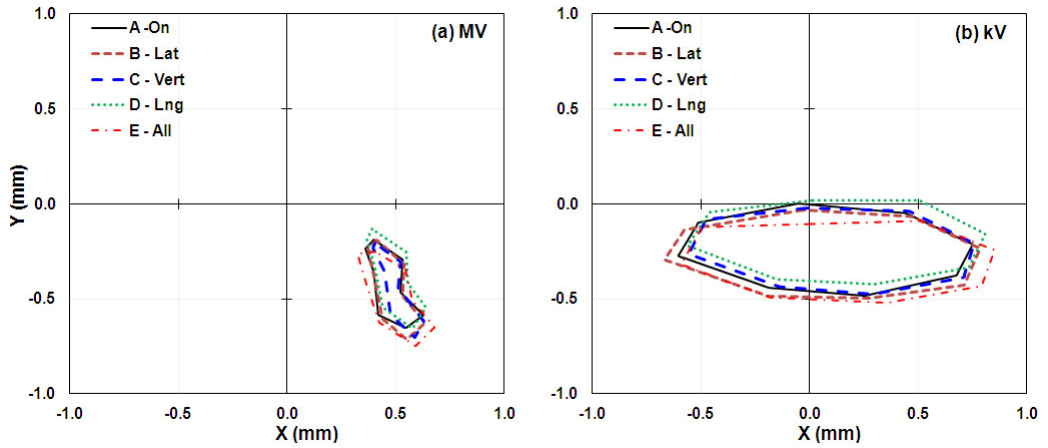
IsoCal results for phantom offset positions showing the lateral (X) and longitudinal (Y) offsets in a full gantry rotation between the MV (left) and kV (right) imager centers and the projected treatment isocenter for four offset phantom positions. A: phantom aligned with room lasers; B: phantom offset about 5 mm laterally; C: phantom offset about 5 mm vertically; D: phantom offset about 5 mm longitudinally; E: phantom offset about 5 mm each of the three directions.

The IsoCal calibration results for the four off‐optimal phantom positions (calibrations B, C, D, and E) were compared with those of calibration A, in which the phantom was set up in the optimal position (Fig. 4). These data indicate that the IsoCal system is not sensitive to the phantom setup. But the IsoCal software does issue a warning on the report screen indicating that the phantom setup exceeds the tolerance range of ±5 mm. We would still recommend that the IsoCal phantom be set up according to the manufacturer's instructions.

### C. Effects of variations in phantom construction

**Figure 5:**
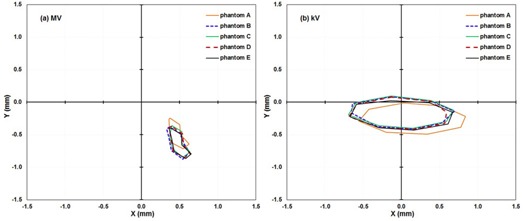
IsoCal results for five different IsoCal phantoms test on the same linac showing the lateral (X) and longitudinal (Y) offsets in a full gantry rotation between the MV (left) and kV (right) imager centers and the projected treatment isocenter for five IsoCal phantom sets A to E.

We studied the effects of variation in the IsoCal phantom/collimator plate construction by performing the IsoCal calibration procedures on the same linac on the same day with five different phantom sets (IsoCal phantom/collimator plate). We found that four of the five phantom sets gave identical results; the first phantom set (phantom A) showed a systematic difference in both kV and MV imager position of a 0.2–0.3 mm (Fig. 5). The IsoCal phantom set that showed the different results was not the one that was purchased earlier than the other phantoms. This variation is about twice as large as the uncertainty noted from repeated calibrations with the same IsoCal phantom set (Fig. 2).

### D. uncertainties in the IsoCal system

A formal analysis of uncertainties for the IsoCal system is beyond the scope of this paper, but we have examined components of the uncertainties by: a) repeated IsoCal calibrations on the same linac with the same phantom set; b) repeated IsoCal calibrations on the same linac with different phantom sets; and c) by intentionally introducing uncertainty into the initial phantom setup position. These tests allow us to estimate the uncertainty: a) from the software's determination of the BB/central pin locations on the images and their effect on the overall results; b) from the variations in phantom/collimator plate construction; and c) from the user's setup of the phantom. The sum of the components of the uncertainties (added in quadrature) is 0.4 mm (Table 2).

**Table 2:** Sources of uncertainties in the IsoCal system

*Component of Uncertainty*	*Estimated Uncertainty (maximum)*
Determination of the locations of BBs/pin in images	0.2 mm (from repeated IsoCal calibrations)
Phantom geometry	0.3 mm (from comparisons across different phantom sets)
Phantom setup	0.2 mm
Total uncertainty	0.4 mm

### E. In‐house, WL‐based method testing

**Figure 6:**
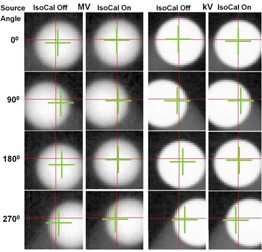
WL‐based BB phantom images with IsoCal correction off and on. From the top row to the bottom row, the source angles are 0°, 90°, 180°, and 270°. The red crosshairs represent the digital graticules in the MV/kV images. The green crosshairs represent the treatment isocenter. Note: the BB for our in‐house WL test, shown in these images, is at a fixed location, not necessarily at the treatment isocenter.

We obtained images for our in‐house WL phantom at the four cardinal gantry angles with and without the IsoCal correction applied (Fig. 6). A comparison of the images with IsoCal corrections applied (“on”) to those without IsoCal corrections (“off”) shows that the position of the digital graticule (red cross) was consistently closer to the treatment isocenter that was determined by our in‐house WL system (green crosshairs) when the IsoCal correction was applied. It should be noted that in the figures, the BB is not at the treatment isocenter because this is not required for our in‐house WL system.

**Figure 7:**
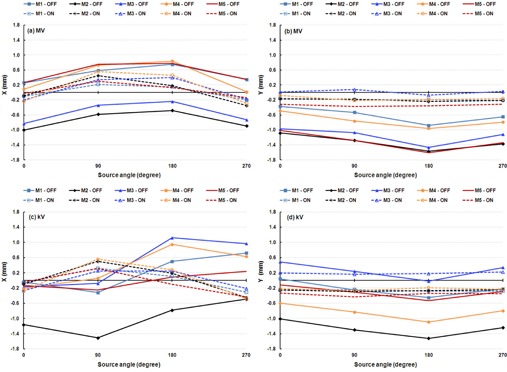
WL determination results for five linacs: (a) MV image, X direction; (b) MV image, Y direction; (c) kV image, X direction; (d) kV image, Y direction. Solid lines indicate that IsoCal corrections were off, and dashed lines indicate that IsoCal corrections were on.

We compared WL calibration results for four cardinal source angles with IsoCal corrections off and on for the five linacs (Fig. 7). The results indicate that when the IsoCal corrections were applied, the offset between the MV and kV imager centers and the treatment isocenter was reduced from greater than 1.6 mm to less than 0.6 mm.

When IsoCal corrections were applied, the shifts determined by the WL method in the X and Y directions in the four cardinal source angles for five linacs were within 0.5 mm for the majority of the test points (Table 3). The largest shift determined by the WL method was 0.6 mm for both the MV and the kV imagers across the linacs when the IsoCal corrections were applied. These results indicate that the agreements between the IsoCal and WL methods are within 0.6 mm.

**Table 3:** In‐house, WL‐determined offsets (mm) between treatment isocenter and electronic graticule (DICOM isocenter in images) with IsoCal corrections applied for the five linacs. Data are scaled to the plane of the isocenter in the lateral (X) and longitudinal (Y) directions in the panel coordinate system

*Source Angle*	*M1*		*M2*		*M3*		*M4*		*M5*	
	*X*	*Y*	*X*	*Y*	*X*	*Y*	*X*	*Y*	*X*	*Y*
*MV*										
0°	−0.1	−0.2	−0.1	−0.2	−0.2	0.0	−0.2	−0.1	0.0	−0.3
90°	0.2	−0.2	0.5	−0.2	0.3	0.1	0.6	−0.2	0.3	−0.4
180°	0.2	−0.2	0.2	−0.2	0.4	−0.1	0.5	−0.2	0.1	−0.4
270°	−0.2	−0.2	−0.4	−0.2	−0.2	0.0	−0.3	−0.2	−0.1	−0.3
*kV*										
0°	−0.1	−0.3	−0.1	−0.3	−0.3	0.2	−0.3	−0.2	0.0	−0.3
90°	0.3	−0.2	0.5	−0.3	0.3	0.2	0.6	−0.3	0.3	−0.4
180°	0.1	−0.3	0.2	−0.3	0.3	0.2	0.3	−0.2	−0.1	−0.3
270°	−0.3	−0.3	−0.5	−0.2	−0.2	0.2	−0.5	−0.2	−0.4	−0.4

### F. VC method testing

**Figure 8:**
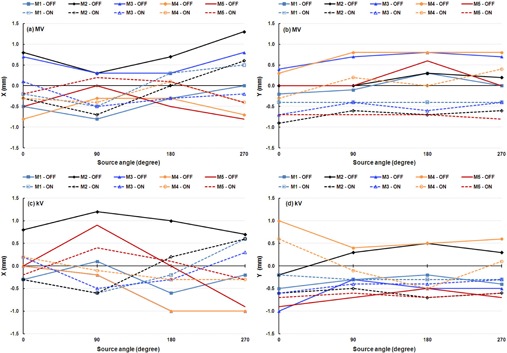
VC determination results for five linacs: (a) MV image, X direction; (b) MV image, Y direction; (c) kV image, X direction; (d) kV image, Y direction. Solid lines indicate that IsoCal corrections were off, and dashed lines indicate that IsoCal corrections were on.

The VC method imager panel offsets were measured without IsoCal corrections (“off') and with IsoCal corrections applied (“on”) for the four cardinal source angles of the MV and kV imagers across the five linacs (Fig. 8). The agreements between those two methods are indicated by the VC offsets with IsoCal corrections applied and have a maximum value of 0.9 mm for the MV imager and 0.7 mm for the kV imager (Table 4). It should be noted that the VC system is the only one of these three systems in which phantom setup is critical. This critical phantom setup is based on either the room lasers or the projection of the crosshairs in the light field, both of which have an uncertainty of 1 mm.

**Table 4:** VC‐determined offsets (mm) between the treatment isocenter and electronic graticule (as displayed on the OBI workstation) with IsoCal corrections applied for the five linacs. The data are scaled to the plane of the isocenter in the lateral (X) and longitudinal (Y) directions in the panel coordinate system

*Source Angle*	*MI*		*M2*		*M3*		*M4*		*M5*	
	*X*	*Y*	*X*	*Y*	*X*	*Y*	*X*	*Y*	*X*	*Y*
*MV*										
0°	−0.2	−0.4	−0.3	−0.9	0.1	−0.7	−0.3	−0.3	−0.2	−0.7
90°	−0.5	−0.4	−0.7	−0.6	−0.5	−0.4	−0.4	0.2	0.2	−0.7
180°	0.3	−0.4	0.0	−0.7	−0.3	−0.6	0.1	0.0	0.1	−0.7
270°	0.5	−0.4	0.6	−0.6	−0.2	−0.4	−0.4	0.4	−0.4	−0.8
*kV*										
0°	−0.3	−0.2	−0.3	−0.6	0.2	−0.6	0.2	0.6	−0.2	−0.7
90°	−0.6	−0.3	−0.6	−0.5	−0.5	−0.4	−0.1	−0.1	0.4	−0.6
180°	−0.2	−0.3	0.2	−0.7	−0.3	−0.4	−0.3	−0.5	0.1	−0.7
270°	0.6	−0.3	0.6	−0.6	0.3	−0.3	−0.3	0.1	−0.3	−0.6

## III. DISCUSSION

Unlike VC phantom‐based^(3)^ and other phantom‐based calibrations,^4^, ^5^, ^6^ the IsoCal calibration method does not require the phantom center to be positioned exactly at the treatment isocenter or mechanical isocenter; instead, the IsoCal algorithm determines the treatment isocenter and calculates the distance between the treatment isocenter and the phantom center, thereby eliminating setup error and minimizing the uncertainty of the calibrations to less than 0.6 mm. The easy setup, high accuracy, and reproducibility of the calibration results make the IsoCal a convenient and efficient tool for initial testing and periodic QA of the geometry of Varian C‐series OBI‐EPID imaging systems.

IsoCal specifies that the tolerance range of the maximum offset of MV and kV imager centers from the treatment isocenter is within 3 mm at the location of the panel (2 mm at the plane of isocenter). Calibration results that are out of the tolerance range (>2 mm at isocenter) indicate that physical adjustments of imager panel(s) are needed. It is also worth noting that this product works differently from the similar system on the Varian TrueBeam linacs. On the TrueBeam platform, the determined offsets are corrected by fine motions of the imaging panels as a function of gantry angle. On the Varian C‐series platform, however, the panel location is not adjusted; rather, the OBI system applies the IsoCal‐determined gantry angle‐dependent corrections to the image position both internally 2D/2D and 3D/3D for matching and in the exported DICOM data as modifications to the X‐Ray Image Receptor Translation tag (3002 000D).

Two independent methods, the in‐house WL method and the VC phantom‐based method, were used to check the IsoCal calibrations. In the WL method, the offsets of the MV and kV imager centers were referenced on the treatment isocenter. However, the WL and IsoCal methods determine the treatment isocenter in different ways. The WL method is essentially based on the field edges defined by the predefined size of collimator apertures at selected gantry and collimator angles.^(10)^ Gantry sag is explicitly accounted for in the WL method.^14^, ^15^ For simplicity, collimator rotation is not considered in our current version of the WL method (we did tested the WL system with three different collimator angles (0°, 90° and 270°) separately; the calibration uncertainty was within 0.5 mm). IsoCal is basically identical in concept to WL method. The collimator in WL is replaced by the collimator attenuating button in IsoCal. WL has a single BB stationary phantom and the location of the BB with respect to the center of the each field can be determined numerically of by the software, whereas IsoCal has 16 BBs and a stationary phantom placed near the isocenter. In both methods, the treatment isocenter is determined relative to that of the phantom by numerical methods. IsoCal takes into account collimator uncertainty and gantry sag in its estimate of the treatment isocenter. The differences in results between WL and IsoCal are experimental and not due to any fundamental difference between the two approaches. For the VC method, accurate calibration results required setting up the phantom center exactly at the treatment isocenter. In actual clinical practice, we set up the VC phantom according to the room lasers, which represent the mechanical isocenter. Ideally, the mechanical isocenter coincides with the treatment isocenter. Any offset of the mechanical isocenter and/or room lasers will affect the accuracy of the VC phantom calibration results. However, the VC phantom calibration method is still considered a valuable method for a quick, intuitive geometric check of the centers of the OBI and EPID imaging systems.

IsoCal calibration reports the phantom center (defined by crosshairs on the phantom) offsets from the treatment isocenter in a 3D vector (X, Y, Z), which indicates the offsets in the lateral (X), longitudinal (Y), and vertical (Z) directions. If we set up the IsoCal phantom center precisely to the room lasers, we can adjust the room lasers to align with the treatment isocenter according to the offsets of the phantom center from the treatment isocenter.

IsoCal calibration also reports the maximum deviation from the central axis of the treatment beam for full gantry rotation. It is similar to the maximum radius of the isocenter in a star shot for the gantry rotation. This value is a metric for the treatment beam uncertainty.

Our independent WL calibrations indicated that the offsets of the MV and kV imager centers from the treatment isocenter were reduced when the IsoCal corrections were applied (Fig. 7 and Fig. 8). These results showed strong evidence of improvement of alignment between the treatment isocenter and the MV and kV imager centers after using IsoCal calibration and corrections. The WL method also provides an independent verification of the IsoCal calibrations that apply to the OBI‐EPID imaging systems for correcting the geometric imperfections of the MV and kV imaging systems.

## V. CONCLUSIONS

This study demonstrates that IsoCal is an accurate and consistent calibration and QA system for geometric calibrations and for verifications of the MV and kV imaging systems. IsoCal shows promise as a convenient, stable, efficient tool for quantitative calibration and evaluation of geometric accuracies of both MV and on‐board kV imaging systems across Varian C‐series platforms. We have, therefore, implemented IsoCal into our monthly QA procedures for Varian C‐series linacs equipped with OBI‐CBCT and EPID imaging systems. Our procedure is to run IsoCal and compare the results with those of the previous applied calibration; if they are consistent, then the previous calibration is retained; if they are different, then the new calibration is applied and cross‐checked with the VC method or another method.

## ACKNOWLEDGMENTS

Peter Balter receives research funding from Varian Medical Systems. The other authors have no conflicts of interest to disclose. We thank Kathryn Carnes and Sarah Bronson for scientific editing of this manuscript.

## APPENDICES

**Appendix A: Theory of the IsoCal Operation**

In the IsoCal calibration process, image analysis software identifies the locations of the BBs and collimator plate pin in the acquired megavoltage (MV) and kilovoltage (kV) images, and geometry analysis software uses the identified BB and pin locations to calculate corrections for any nonideal source plus imager geometry.

### A. Identifying location of objects (BBs, pin) in the projection images

The process of finding the 2D coordinates of the BBs on the cylindrical phantom and the steel pin on the collimator plate in the projection images is divided into inspection and tracking. The inspection identifies the location of the BBs in only the first projection image, and tracking identifies the location of the BBs in subsequent images. The information for the first image can be used to speed up processing considerably for subsequent images. As a result of the track phase, each image is reduced to an accurate set of 2D BB locations. The upcoming analysis phase can now occur without any more reference to the images themselves.

### B. Computation of geometric parameters

#### B.1 Coordinate systems

There are two main coordinate systems used in the algorithm: 1) the fixed coordinate system, which is stationary with respect to the room, whereby once the IsoCal BB phantom is placed on the couch, it is stationary in the fixed coordinate system throughout the kV and MV scans; 2) the gantry coordinate system, which is stationary with respect to the gantry and which moves when the gantry rotates. Note that there is a known (but different) transformation between the gantry coordinate system and the fixed coordinate system at every gantry angle. In this coordinate system, the IsoCal BB phantom appears to rotate.

#### B.2 Positions and orientations of the phantom

The position and orientation of any rigid object can be described by six degrees of freedom (6DoF) coordinates (X, Y, Z, roll, pitch, and yaw). In the analysis process, the 2D coordinates of the projections of the actual BBs onto the imager are calculated and compared with the measured BB coordinates obtained in the track phase, and then the parameters for a fitting function, which is fit to a series of measurements, can be determined. The process first determines the parameters of the source‐imager system for every gantry angle in the fixed coordinate system and calculates average SID values for all acquired images. Then, using the average SID, the 6DoF parameters of the calibration phantom are found for every gantry angle in the gantry coordinate system; the imager and source appear to be stationary, and the IsoCal BB phantom appears to be rotating. The result is a complete set of positions and orientations of the phantom at each gantry angle.

#### B.3 Determination of rotation center of the imaging systems

**Figure A.1.**
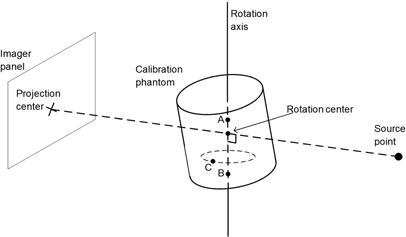
Geometry for the rotation center estimation. All parameters are calculated in the gantry coordinate system; thus, the phantom appears to be rotating. Two fixed points in the phantom, A and B (not necessarily BB locations), are identified as points with no transverse sinusoidal motion; these define the rotation axis. Point C is an example of a point that will be rejected as a possible rotation axis point because it moves in sinusoidal motion as the phantom rotates. Note that the rotation axis need not correspond with the symmetry axis of the cylinder. The rotation center and projection center can be calculated from the rotation axis and the location of the source point.

Once the 6DoF parameters of the phantom are found for each projection, the algorithm finds two fixed points within the phantom that are on the rotation axis of the phantom (Fig. A.1). Since the 6DoF parameters of the phantom are known at every gantry angle, the software can determine the position of any given point within the phantom, with respect to the gantry coordinate system, as a function of gantry angle. Once points A and B (Fig. A.1) are determined, they define the rotation axis of the rotating phantom. The intersection of the rotation axis and the line perpendicular to the axis going through the source point location is designated as the rotation center. Note that the information from every gantry angle is used when determining the rotation axis and the rotation center. Therefore, only one rotation axis and one rotation center are computed for each input data set.

The source‐to‐axis distance (SAD) is then determined as the distance between the rotation center and the source point.

The projection of the rotation center onto the imaging panel at each gantry angle is then found. This projection defines the projection center. Note that one projection center is determined for each gantry angle.

#### B.4 Radiation treatment isocenter determination

**Figure A.2.**
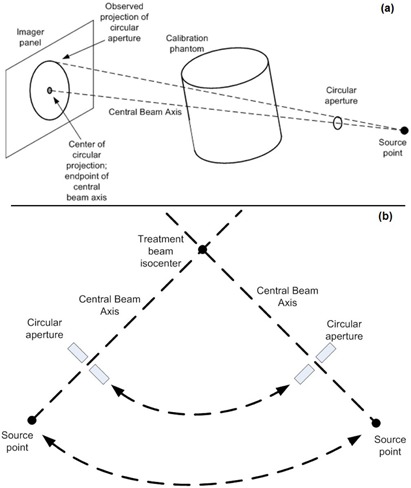
Determination of the central beam axis (a): the circular aperture in the figure represents the semi‐radiopaque pin located on the collimator plate. Determination of the treatment isocenter (b) showing the gantry rotation axis points out of the paper. The treatment isocenter is the intersection of the central beam axes from multiple gantry angles. The actual computation takes the best‐fit intersection from eight gantry angles, but in the figure only two are shown.

The first step in identifying the radiation treatment isocenter is to find the central axis of the MV beam. Four images are acquired with four collimator angles, and the exact center of the pin in the four images is located. An algorithm then determines the best‐fit circle that passes through all the identified pin locations. The center of the best‐fit circle is taken as the central axis of the MV beam (Fig. A.2(a)). The offset between the exact center of the pin and the calculated central axis of the MV beam for a given collimator angle is used for subsequent computations of the location of the treatment isocenter.

The central axis for the MV beam at each gantry angle is initially computed in its own gantry‐based coordinate system at its own gantry angle and, therefore, all the central axes need to be converted to a common coordinate system if their intersection is to be computed. The previously computed 6DoF parameters of the phantom at every gantry angle are used to transform the axes from the individual gantry‐based coordinates to a common fixed/room‐based coordinate system. Finally, a subset (usually eight) of these (now room‐based) axes are taken, and the bestfit intersection of the subset of axes is determined (Fig. A.2(b)). The intersection is computed as the point that minimizes the sum‐squared distance to each of the central axis trajectories.

#### B.5 Shift vector computations

The IsoCal software uses the geometric information obtained from the image analysis to calculate the actual positions of the MV and kV imager isocenters with respect to the treatment isocenter as a function of gantry angle correcting for small misalignments in the imaging panels and the mechanical sag of these devices. These data are reported to the user as a graph showing the imager rotation shift vector, which is the distance between the projection of the treatment isocenter and the rotation center of the imager onto the images as a function of gantry angle. These data are also written into an XML file that can be used by the OBI application to apply these corrections.
